# Socio-Cognitive Engineering of a Robotic Partner for Child's Diabetes Self-Management

**DOI:** 10.3389/frobt.2019.00118

**Published:** 2019-11-15

**Authors:** Mark A. Neerincx, Willeke van Vught, Olivier Blanson Henkemans, Elettra Oleari, Joost Broekens, Rifca Peters, Frank Kaptein, Yiannis Demiris, Bernd Kiefer, Diego Fumagalli, Bert Bierman

**Affiliations:** ^1^TNO Perceptual and Cognitive Systems, Soesterberg, Netherlands; ^2^Department of Intelligent Systems, Delft University of Technology, Interactive Intelligence, Delft, Netherlands; ^3^IRCSS Ospedale San Raffaele, Center for Advanced Technology in Health and Wellbeing, Milan, Italy; ^4^Department of Electrical and Electronic Engineering, Imperial College, London, United Kingdom; ^5^DFKI, Saarbrucken, Germany; ^6^Mixel, Milan, Italy; ^7^Produxi, Zeist, Netherlands

**Keywords:** child-robot interaction, conversational agent, human-robot partnership, socio-cognitive engineering, diabetes management, personal health, pervasive lifestyle support

## Abstract

Social or humanoid robots do hardly show up in “the wild,” aiming at pervasive and enduring human benefits such as child health. This paper presents a socio-cognitive engineering (SCE) methodology that guides the ongoing research & development for an evolving, longer-lasting human-robot partnership in practice. The SCE methodology has been applied in a large European project to develop a robotic partner that supports the daily diabetes management processes of children, aged between 7 and 14 years (i.e., Personal Assistant for a healthy Lifestyle, PAL). Four partnership functions were identified and worked out (joint objectives, agreements, experience sharing, and feedback & explanation) together with a common knowledge-base and interaction design for child's prolonged disease self-management. In an iterative refinement process of three cycles, these functions, knowledge base and interactions were built, integrated, tested, refined, and extended so that the PAL robot could more and more act as an effective partner for diabetes management. The SCE methodology helped to integrate into the human-agent/robot system: (a) theories, models, and methods from different scientific disciplines, (b) technologies from different fields, (c) varying diabetes management practices, and (d) last but not least, the diverse individual and context-dependent needs of the patients and caregivers. The resulting robotic partner proved to support the children on the three basic needs of the Self-Determination Theory: autonomy, competence, and relatedness. This paper presents the R&D methodology and the human-robot partnership framework for prolonged “blended” care of children with a chronic disease (children could use it up to 6 months; the robot in the hospitals and diabetes camps, and its avatar at home). It represents a new type of human-agent/robot systems with an evolving collective intelligence. The underlying ontology and design rationale can be used as foundation for further developments of long-duration human-robot partnerships “in the wild.”

## 1. Introduction

Despite substantial progress in AI, robotics, conversational agents, and related technologies (Klopfenstein et al., 2017; Wu et al., 2017; Anjomshoae et al., 2019; Montenegro et al., 2019), social or humanoid robots do hardly show up in sound long-term field studies for pervasive human benefits such as child health (Moerman et al., 2018; Dawe et al., 2019; Robinson et al., 2019). The studies with a prolonged deployment and long-term behavior support ambition have been more exploratory, covering “only” a few robot functions and interactions in a time span of a couple of weeks, for example to explore child-robot relationship development (Looije et al., 2016; Westlund et al., 2018). To make this ambition reality within the foreseeable future, the research & development approach has to change substantially: We have to take an integrative socio-cognitive approach in which robots are researched and developed as part of a human-robot collective that has collaborative intelligence (Epstein, 2015; Johnson and Vera, 2019; Rahwan et al., 2019).

This paper presents such an approach, the socio-cognitive engineering (SCE) methodology that aims at such a developing collective: The building of human-robot partnerships for prolonged performance and well-being. In an extensive case study, the European Personal Assistant for a healthy Lifestyle (PAL) project, this methodology has been applied to develop a robotic partner and human-robot activities that support the daily diabetes management processes of children, aged between 7 and 14 years (i.e., supporting a healthy lifestyle).

Type 1 Diabetes Mellitus (T1DM) is one of the main chronic diseases in childhood with severe consequences for physical and mental well-being. The disease prevalence is rising substantially, doubling every 20 years. T1DM is often diagnosed in early or middle childhood (age between 1 and 11 years) based on symptoms of high or low blood glucose (i.e., hyper- or hypoglycemia) (Betts et al., 1996; Boyer and Paharia, 2008; Jin et al., 2017). Symptoms of a hyper can be headaches, fatigue, thirst, and nausea, while a hypo can start with tremors, sweating and palpitations, and eventually can continue in confusion, impaired thinking, and even seizures. The long-term health consequences of T1DM can be serious, damaging the eyes (retinopathy), peripheral nerves (neuropathy), or kidney (nephropathy) (Centers for Disease Control and Prevention, 2011). Managing T1DM requires strict lifestyle adjustments, which proves to be complex and demanding (Iannotti et al., 2006). Daily management behaviors are, for example, monitoring blood glucose (at least 4 times a day), counting carbohydrates before every meal or snack, anticipating physical exercises, and calculating and administering insulin (Boyer and Paharia, 2008). When children enter puberty, the management challenges are increasing: Bodily changes (e.g., hormones) bring about new dynamics in the blood glucose regulation processes, socio-emotional changes bring about different (possibly negative) appraisals, and autonomy development can bring about resistance to parents and caregivers advises. Unfortunately, most children are not, in advance, well-prepared or -trained to deal with these challenges, as the parents can take care of them well. The result is a decrease in glycemic control and regimen adherence when children enter puberty (Ellis et al., 2007; Pai and Ostendorf, 2011).

We started the PAL project to develop a social robot that supports the child in learning to correctly manage T1DM and, this way, prevents serious consequences to appear at the age of puberty. The envisioned robot acts as partner in a (small) diabetes management team, primary for the child (as a “pal”), but also for the Health Care Professional (HCP) and parent (as a “mediator,” e.g., for responsibility transfer from parent to child). It is a conversational agent that is integrated into a distributed behavior change support system, embodied as a humanoid robot in hospitals and diabetes camps, and as an avatar on a tablet at home. Via a mobile timeline and dashboards, the diabetes management activities, information processes and outcomes are visible, accessible, and manageable at all locations. The collective Human-PAL intelligence is evolving over time based on (1) the incremental additions and refinements of robot capabilities in the successive development cycles and (2) the intrinsic learning capabilities of the humans and robots (e.g., based on experiences and feedback).

This paper provides an overview of the PAL research & development activities and outcomes, focusing on three general research questions. The first question is: “How to develop human-agent partnerships for long-term lifestyle support?” The second question concerns the design outcome: “How can a robotic partner support the daily diabetes management of children over a longer period?” The third evaluative question is: “Does this partnership improve child's diabetes-control and well-being?” Section 2 argues that the SCE-methodology provides an answer to the first question, and provides an overview of this methodology. Section 3, 4, and 5 describe the application and results for each SCE-component: the foundation, specification and evaluation of the PAL system. Taken together, they present the evolving knowledge base and partnership behaviors of the human-robot collective to be applied and further developed in practice. Section 6 contains the general discussion and conclusions.

## 2. Socio-cognitive Engineering

In the eighties, cognitive (system) engineering was proposed to integrate social sciences, like cognitive psychology, into the design of human-machine systems or so-called *joint* cognitive systems (Norman, 1986; Woods and Roth, 1988; Rasmussen et al., 1994; Hollnagel and Woods, 2005). Subsequently, this approach was refined to facilitate re-usability and theory building (generalization) by the construction of a design rationale that explicates the contextual dependencies, calling the methodology *situated* cognitive engineering (SCE) (Neerincx and Lindenberg, 2008; Neerincx, 2011). This rationale describes the design solution with its theoretical and empirical foundation in a coherent and concise format and structure. Core is the specification of claims (“hypotheses”) on the effects of machine (e.g., robot) functions in specific use cases, and the development of design patterns for the corresponding machine behaviors (Neerincx et al., 2016b; Looije et al., 2017). To better address the technological progress on Artificial Intelligence (AI), Robotics, Conversational Agents and Connectivity, with their capabilities to transform social processes and human-technology relationships, SCE toke a more principled focus on human-agent/robot teamwork and patterns, and got its corresponding new first syllable: Socio-Cognitive Engineering (Sharples et al., 2002; Bradshaw et al., 2012; Van Diggelen et al., 2018). In our view, SCE can contribute to the research and development of the robotic systems by supporting the acquisition, modeling, sharing, and extension of the evolving social intelligence.

Long-term interaction “in the wild” is an important research and development challenge for socially assistive and educational robots (SAR). For example, Coninx et al. (2016) stated that, to pursue learning and therapeutic goals through child-robot interaction, it is important to ensure the child remains engaged in the relationship and that the child experiences progress in achieving educational goals. To establish such engagement and to accommodate individual differences, they developed an adaptive social robot with which children can perform various activities. This robot was evaluated in three 1 h hospital sessions (with about 2 weeks in between each session), showing positive effects on engagement and bonding (Looije et al., 2016). The design rationale was well-explicated by Looije et al. (2017), but did hardly include formal specifications of robot's social intelligence and did not inform *how* to extend. As a second example, Jones and Castellano (2018) used an open learner model (OLM) for a robotic tutor that promotes self-regulated learning (SRL) in a personalized scaffolding process. Based on this model, the robot shows skill meters for each competency, prompts the learner to reflect on their developing skills, and can suggest to work on an activity of an appropriate difficulty level for learning. The robot was evaluated at a primary school during 4 sessions (1 session per week) with positive results. As far as we know, the underlying OLM- and domain-models are not formalized in a way that enables (automatic) reasoning on causes and effects of the robot feedback (as an evolving “social educative intelligence”). Gordon et al. (2016) provide a third example of long-term human-robot interaction in which children play a second-language learning game with a “social robotic learning companion.” An affective policy was developed to provide appropriate affective responses when the child finished a task or was not active for a while. The robot was evaluated in preschool classrooms for a duration of 2 months (each child interacted from 3 to 7 sessions). Personalization of the affective response had a positive effect on child's emotional state (valence). This study is a good example of the design of model-based social responses, but the scope is still rather limited and does not (yet) address robot's role in the class room (e.g., its relation with the teacher). As a last example, Clabaugh et al. (2018) presented preliminary results of a 30-day, in-home case experiment with a robot for children with autism. Their findings underline the importance of personalization of robots and show the relevance of research in realistic long-term, family-situated contexts. For example, parents were more comfortable to let the children interact with the robot independently and reported that the robot gave them more time for other things. How the robot could systematically support such situated social processes is not yet clear, however. Findings of these studies underline the relevance of applying a comprehensive socio-cognitive methodology that systematically addresses the social context, the building of a shared human-robot knowledge base and the opportunities to improve and learn continuously.

**Figure 1:**
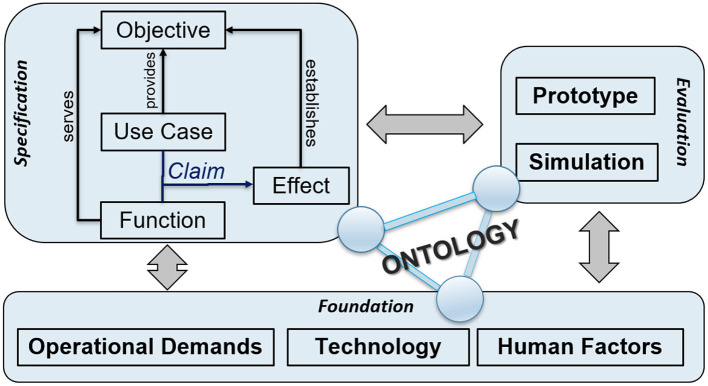
Overview of the socio-cognitive engineering methodology.

Figure 1 presents an overview of the Socio-Cognitive Engineering (SCE) methodology, distinguishing the foundation, specification, and evaluation. To establish the *foundation*, i.e., the operational demands, technology and human factors, a selection of established human-computer interaction and human factors methods can be applied, e.g., from the People, Activity, Context and Technology (PACT) analyses (Benyon, 2019) or Cognitive Work Analyses (Vicente, 1999; Naikar, 2017). SCE puts specific emphasis on the identification of expert knowledge and cognitive theories that are relevant and can be formalized for implementation in the human-robot knowledge-base. See, for example, the “situated design rationale” method for formalizing and contextualizing behavior change support techniques of Looije et al. (2017). From the foundation, a design *specification* is derived that defines “what” the system shall do (function) in a set of use cases (“when”) to bring about a desired effect (i.e., the claim, “why”). In the *evaluation*, the claims are tested via prototyping or simulations, in order to validate and refine the foundation and design specification. It is an iterative, incremental development process, aiming at a sound, theoretically and empirically grounded, prototype with a coherent description of its design rationale. Each design-test cycle will advance (a) the prototype, (b) its foundation in the human factors, technology and operational demands, and (c) the design specification. For the building, maintaining and re-using of design knowledge, SCE distinguishes the following development principles. First, creating human-centered AI and robots is viewed as an inter-disciplinary collaborative activity with active stakeholder involvement during the complete development process (cf. Riek, 2017). Second, functional modules are defined and tested incrementally in an iterative refinement process. As learning and adaptation are key characteristics of human-AI systems, this process of iterations should continue during the complete life-cycle of these systems. Third, design decisions are explicitly based on claims analyses, explicating the up-downside trade-offs. Fourth, keeping and sharing the design rationale is key for progress and coherence in the development of AI and social robots. Fifth, a common ontology should be developed and implemented, which defines the core concepts, with their relationships, for human-robot collaboration (e.g., tasks) and communication (e.g., style).

The PAL project applied these five principles in the three design-test cycles in Italy and the Netherlands (in a period of 4 years). In each cycle, we constructed, extended and refined the foundation, design specification and prototype of the PAL system. It should be noted that the direct stakeholders (children, parents, and Health-Care Professionals), the designers & engineers and the researchers (from computer science, AI, Psychology, educational science, health-care, human-computer interaction) were actively involved in the PAL research & development team from the start to the end of the project. The next sections provide more information on the SCE theories, models and methods that were applied in the PAL project.

## 3. Foundation of Robotic Lifestyle Partner

### 3.1. Human Factors

Human Factors theories and methods should be used in the development of robotic lifestyle partners. The *Self-Determination Theory* (SDT) provides a coherent and well-founded starting point to support the behavior change that disease management requires. It distinguishes three human basic needs that affect the development and habituation of human behaviors in a social environment: The needs for competence, autonomy and relatedness (Legault, 2017; Ryan and Deci, 2017). By supporting these needs, as important sub-objectives, PAL is expected to achieve the main objective of enhanced self-management. For each basic need, a support strategy for a social robot (NAO) has been designed and tested successfully for children with diabetes (Blanson Henkemans et al., 2017a).

First, autonomy proves to be supported by providing choice and rationale for the (educative) activities, acknowledging children's feelings and minimizing pressure and control. It is expected that personalizing the learning objectives and providing explanations improves the responsibility transfer further. The difficulty of the learning tasks should be attuned to the skill level of the learner for an optimal learning experience and outcome. The Zone of Proximal Development (ZPD) theory states that adaptive support (or “scaffolding”) can establish the required balance, encouraging and advancing the individual learning processes (Vygotsky, 1980; Chaiklin, 2003; Charisi et al., 2015). Such a balance will also help to develop an adequate level self-efficacy (Bandura, 1977).

Second, competence proves to be enhanced by providing effectance-based (instead of norm-based), reinforcing and challenging feedback. Applying motivational interviewing techniques can help to improve this feedback, i.e., providing appropriate informative feedback (corrective, descriptive, evaluative, or confirmatory responses) and motivational feedback (encouragement, praise, remark, or mood matching) (Schunk and Lilly, 1984; Tudge et al., 1996).

Third, relatedness proves to evolve positively by approaching the child in a personal, positive and respectful way. Via experience sharing in the form of reciprocal disclosures, relatedness can be further enhanced (see the Social Penetration Theory, Cohn and Strassberg, 1983; Altman and Taylor, 1987; Rotenberg and Chase, 1992; Burger et al., 2017).

**Figure 2:**
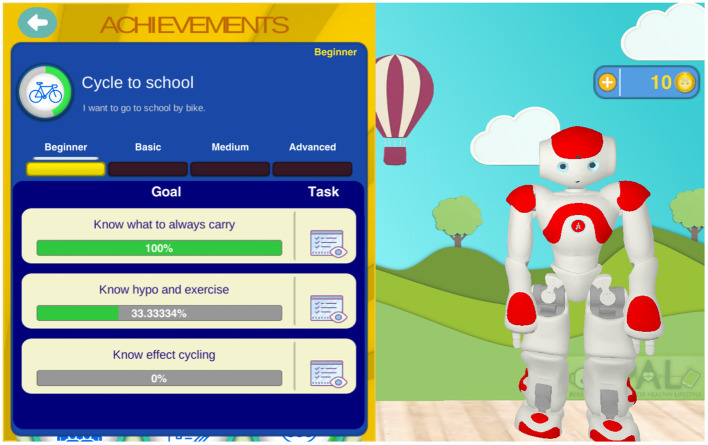
Screenshot of the achievement dashboard in the MyPAL application to follow own goal attainment (this example shows child's progress on “Cycle to school”).

**Figure 3:**
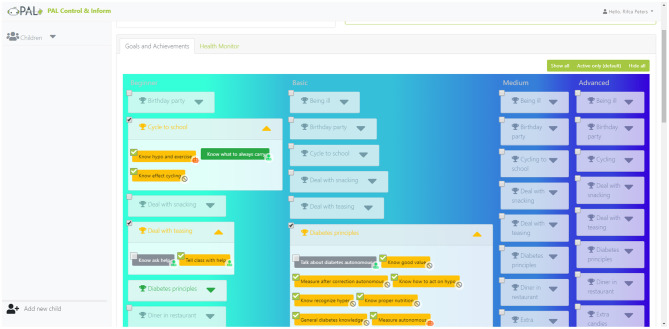
Screenshot of the goal-tree in the PAL control & inform application to select personal goals.

**Figure 4:**
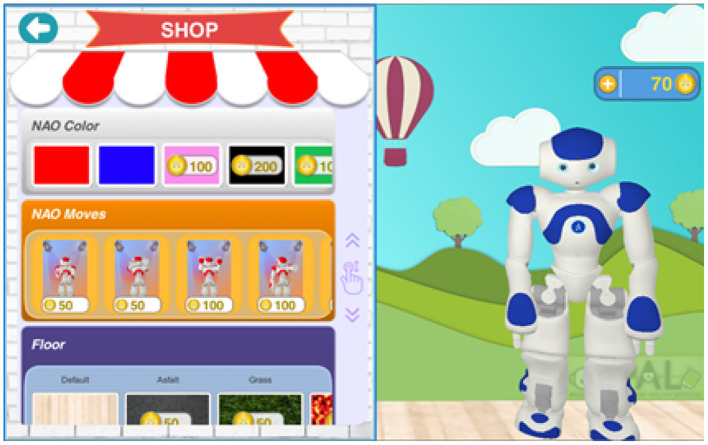
Screenshot of the shop in the MyPAL application where one can buy nice skins and robot dance features with coins earned by doing diabetes related activities in the MyPAL applications.

*Gamification* principles have been studied, proposed, and applied for diverse behavior change support systems, to enhance users motivation, for example for child's diabetes self-management (Blanson Henkemans et al., 2017b). For PAL, we worked out these principles in the following educational games. A quiz is used to learn and test knowledge. A break-and-sort game is used to train the players to rapidly recognize the content of box (e.g., the categories of certain foods), challenging player's reflexes. A memory game provides a relatively relaxed and slow-paced experience for thinking and reflection. The general gamification approach entails an activity-based reward system which enables the “players” to unlock additional features for personalized engaging tasks in the PAL timeline. An achievement dashboard (Peters et al., 2019b) shows the personal achievements and (learning) goals, progress toward attainment and the possible activities for further advancement. The achievements and goals are chosen collaboratively between the child and health care professional (HCP) and selected via the HCP-dashboard (inspired by ability trees). Goal attainments are rewarded with coins. Figures 2, 3 show, respectively, a screenshot of the achievement dashboard and the goal tree. Coins can be earned and used to unlock new desirable content. In PAL four categories were implemented: Floor images, background images, color of the avatar, and dance moves (i.e., features to design dances that can be shown by the avatar and the robot). In a shop, these features can be unlocked and activated. See Figure 4 for a screenshot of the shop in the MyPAL application.

Children are frequent users of interactive technologies for different kinds of purposes, but have hardly been involved in the design process itself to provide their specific needs and ideas (Druin, 1999, 2002; Davis, 2010). A coherent and concise set of *co-design* methods is needed, which (a) allows children to choose their own way of expression and communication and (b) provides complementary insights in their values, needs and situations (Darbyshire et al., 2005). To fulfill this need, we developed the Co-design for Child-Computer Companionship (4C) suite (Blanson Henkemans et al., 2016), consisting of two methods for eliciting daily experiences, needs and values regarding T1DM (i.e., photo-elicitation and user journey map), and three methods for collecting envisioned interactions and requirements for the PAL system (draw-write-tell, story telling, and image theater). The 4C suite has been developed by a multi-disciplinary team, involving robotics researchers, service designers, psychologists and ethicists, to establish a comprehensive, responsible and practical approach for (a) value, need and context analyses, and (b) generation of design ideas. Blanson Henkemans et al. (submitted) provide more background information on the C4 suite and its development.

### 3.2. Operational Demands

The direct stakeholders, particularly the children, parents, and health care professionals (HCPs), were intensively involved in both the design and test activities. At the start of the project, focus group sessions with the HCPs and diabetes organizations provided information on diabetes management and the child, family and context factors, *and* on the support needs of the HCPs themselves. Similarities and differences between the nations and hospitals were identified, and explicated in (a) flow charts of the care processes, (b) descriptions of personas and (b) journey maps for these personas (“disease management related activities of a child and his or her caregivers during the week”).

Every year, the national patient organizations set-up so-called diabetes camps in Italy and the Netherlands, among other things to acquire further insight in children's values, needs and ideas for PAL support, and to assess interim designs and prototypes. As parents were partially present, their values, needs, ideas, and assessments could be acquired too. Follow-up focus group sessions with the HCPs and diabetes organizations provided further information on diabetes management and the corresponding child, family and context factors, *and* on the support needs of the HCPs themselves.

In these sessions, we acquired so-called *value* stories for each direct stakeholder (i.e., child, parent, and HCP), as a first step of the requirement analysis. Value stories have the following format: As stakeholder I want/need requirement to support value in a certain situation. An example is: As a child I need a personal robot that shares experiences by active listening and telling about itself in a similar way to support relatedness in the PAL-activities at the camp, hospital and home. In addition, possible value tensions were identified (such as the tension between privacy and health for the sharing of information about diabetes regime adherence). To address these tensions adequately, we formulated a general requirement on the creation, activation, and adaptability of agreements (permissions, prohibitions, and obligations to share information, e.g., when the child is staying with a friend; cf. Kayal et al., 2018a,b).

### 3.3. Technology

The PAL project uses a state-of-the-art *humanoid robot*, the NAO of Softbank Robotics, which has four microphones, two speakers and two video cameras. The robot is present at the hospital and diabetes camps (and might sometimes visit the child at home or school). As an avatar, a virtual 3D robot model (i.e., a “copy”) was developed in the Unity environment, which has the same appearance, movement and interaction characteristics (there is one “expression model” for these two embodiments). The avatar is developed for Android mobile devices, particularly for a tablet that is used at home.

*Cloud computing* is used to establish an evolving modular and distributed intelligence that facilitates long term interaction (Kehoe et al., 2015). It enables (a) accessing external libraries for enriching interaction such as dialogues, (b) relatively heavy computations such as statistical analyses of previous behaviors and their outcomes, (c) collective human-agent learning (the human and the robot can, in real-time, learn from each others' interactions by means of data sharing), and (d) monitoring the robot's interactions and adapting the decision making where and when needed. The PAL “brain” is set-up in a modular manner to support incremental development [easy addition and updating of (sub-)modules]. All messages go through a common messaging board called the *nexus*. Each module sends messages of a particular type and decides itself to subscribe to specific message types of other modules.

A *hybrid AI* approach was chosen that combines symbolic reasoning methods [like Belief-Desire-Intention (BDI) agent frameworks] with Machine Learning methods. The symbolic reasoning frameworks allow to implement expert knowledge into the system, and to provide meaningful control and interpretable output for the human. The machine learning methods allow to leverage the available data for potentially continuous performance improvements. For example, estimating child's knowledge level is an important continuous process (“user modeling”) for the planning of the next (learning) tasks. Concerning machine learning, a combination of collaborative filtering, Gaussian processing, and covariance matrices is used to track child's knowledge level in PAL (see Cully and Demiris, 2019), and a deep learning Gated Recurrent Unit (GRU) model for aspect extraction to track child's emotional state on the topic of a textual expression (Haanstra and de Boer, 2019). Concerning symbolic reasoning, a Cognitive Agent Architecture Framework is used to provide adaptive—goal-, belief-, emotion-based—explanations (Kaptein et al., 2016; Neerincx et al., 2018), and a dialogue management framework for the human-agent conversations in general.

The knowledge base of the symbolic reasoning framework of PAL entails a *federated ontology*. Ontologies provide explicit, formal descriptions of objects and concepts (their properties), and of the relations among them (Gruber, 1993). In SCE (see Figure 1), the ontology covers concepts from the foundation, specification and evaluation, and functions as an evolving knowledge base that: (1) provides an unambiguous vocabulary and communication between stakeholders, (2) supports system implementation of knowledge-based reasoning functionalities, and (3) serves as a basis for interoperability in human-agent interaction (as they contain human expert knowledge and have an inbuilt logic that machines can process and interpret). The PAL ontology integrates individual ontologies (“models”) via one top-level ontology. These models are high-level building blocks that contain smaller, more specific areas of interest (“frames”) (Neerincx et al., 2016a). When useful, existing ontological frames can be rather easily included in the evolving ontology. The PAL system uses an extended Resource Description Framework (RDF) storage component and reasoner (HFC) to process the knowledge models (classes) and running instances in conjunction (van Bekkum et al., 2016).

Kaptein et al. (in review) provide more background information on the PAL system architecture and technology.

## 4. Specification of situated human-robot partnerships

Based on the human factors, operational and technological analyses of section 3, we worked out the core functions (4.1) and knowledge-base (4.2) of the robotic partner with the corresponding interaction design (use cases, requirements, and claims; 4.3) of the PAL system.

### 4.1. Partnership Functions

**Figure 5:**
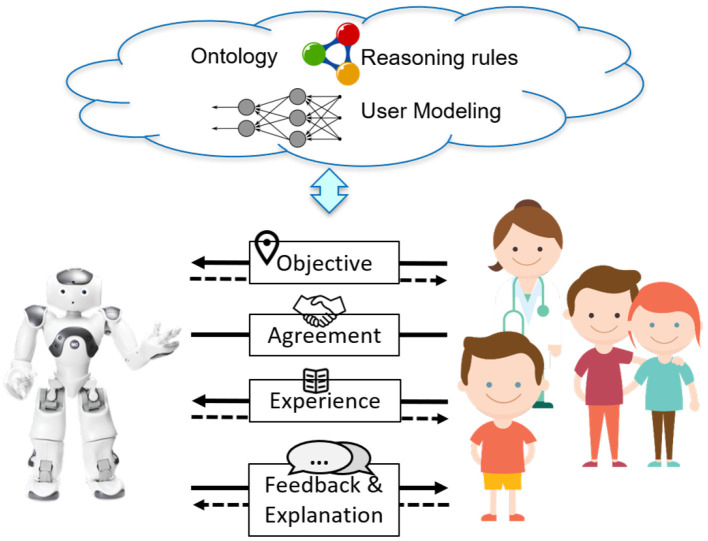
The PAL actor (robot and avatar), its core functions and knowledge-base to act as partner in the diabetes management of a child and his or her diabetes care team.

Five high-level (“core”) functions of a robotic lifestyle partner like PAL are expected to enhance the disease self-management, distinguishing 4 partnership functions in italics (see Figure 5):

1. Providing personal, reliable and reinforcing assistance on diabetes management via learn-by-playing activities.
2. Planning and pursuing joint *objectives* for the disease management. These objectives (like enhanced diabetes management) drive robot activities in a consistent and transparent way, and are compliant with stakeholder values. Furthermore, the style of communication is harmonized with the joint objectives (e.g., showing “warmth,” “competence,” and “dominance,” Peters et al., 2015, 2017b, 2019a).
3. Proposing and committing to *agreements* for value-sensitive information sharing. To address value trade-offs adequately, information sharing might be permitted, prohibited or obliged for specific stakeholders, situations and periods (such as keeping emotional statements private in specific situations).
4. Sharing *experiences* via disclosures that match the disclosures of its human partner. For long-term lifestyle partnerships, mutual understanding and relationship building is crucial (such as learning to cope with the effects of specific stress events and sport activities on the personal blood glucose regulation).
5. Providing *feedback* on partner's behaviors, learning progress, and *explanations* of own behaviors. These responsive and pro-active communications should be constructive and personalized to establish prolonged motivation, learning and trust.

### 4.2. Partnership Knowledge-Base

We worked closely together with health care professionals to obtain and implement an ontology that contains the relevant knowledge and content for these core partner functions. The PAL ontology integrates individual ontologies (“models”) via one top-level ontology. Relevant existing ontological frames were identified and included in the PAL ontology. For some, only parts of the frame were relevant, and therefore partially included (e.g., the self-management activities of diabetes, but *not* the entire professional medical diagnosis and treatment model of diabetes). Other frames had to be extended with additional concepts into a PAL model [e.g., the well-known task ontology Van Welie et al. (1998) in the PAL Objective Model]. The PAL ontology contains models that capture mutually different knowledge; no direct dependencies have to be specified for the concepts of one model to the concepts of another model. The independence of the models has as advantage that it provides clean sub-ontologies which can be reusable in other projects and/or domains. Besides that, this structure had a practical advantage that different project partners could work on the sub-ontologies simultaneously, without interfering with each other. Currently, the PAL ontology consists of ten models, the top-level ontology and nine models that capture different knowledge (most are available as separate files using the Web Ontology Language, OWL, which can be directly re-used in other hybrid AI systems):

- The *PAL Objective Model (POM)* entails a decomposition of achievements into (learning) goals, which are further decomposed into (learning) tasks (Peters et al., 2017a, 2019b). When the underlying tasks are completed, the goal is attained (it can be that *either* task A *or* task B has to be completed). When the underlying goals are attained, the achievement is gained. For example, to gain the achievement of competence for a sleepover, the child has to attain the learning goals to know “how and when to measure blood glucose” and “what to do when I am experiencing some tremors” by completing the corresponding tasks of the diabetes quiz and memory game.
- The *Domain* model describes characteristics of diabetes, the PAL system, the direct stakeholders (end-users) and locations (such as hospital, diabetes camp, home). The classes could be core domain concepts (e.g., actor, activity, food, .) or relate to other classes (e.g., “pen” and “pump” are sub of “device”). Specific information for the dialogue modeling has been included in the domain model (i.e., the classes and properties that constitute the information state of the dialogue components).
- The *Episodic Memory* model combines the Ontology-based Unified Robot Knowledge (OUR-K) with a temporal episode ontology that models the 5W1H (When, Where, Who, Why, What, and How) (Lim et al., 2011, 2013; Han et al., 2013). This model is used to record and activate memory episodes. Based on rules, each episode is tagged with possible triggers (e.g., child login) that, for example, can activate a corresponding speech act.
- The *Agreement* model specifies the underlying concepts with their relations: Type (permission, prohibition, obligation), creditor, debtor, antecedent, consequent, condition, and adaptability (Kayal et al., 2018a,b; Mioch et al., 2018). In the current PAL system, the focus is on the sharing of child's data. For indications of serious health risks (e.g., very high or low blood glucose values, or a long-lasting negative emotional feeling), policies to inform parent and HCP were specified and implemented. The default setting for other information is: Sharing information with PAL is permitted, whereas it is prohibited with other stakeholders (e.g., parent and HCP). Agreements can be set-up to change this information sharing. PAL will act according to these agreements and provide the information as agreed upon.
- The *Semantics* model is tailored to the specific needs of the PAL games (quiz, break & sort, and memory game). We developed a simple frame semantics that is oriented along thematic roles, and deviated from the FrameNet Frame semantics (which would require heavy modification and extension as it is very general). Among other things, it aims to underpin natural language generation and interpretation, and to support multilingualism (i.e., linking concrete realizations in the different languages to the abstract concept as, e.g., Multilingual WordNet does).
- The *Affect* model is based on James Russell's Circumplex Model of Emotions, the Schachter-Singer theory of emotion and Joseph Forgas' Affect Infusion Model (Schachter and Singer, 1962; Forgas, 1995). It describes how Mood and Emotion continuously influence each other.
- The *Interaction Style* model is based on Leary's Interpersonal Circumplex, the Model of Interpersonal Teacher Behavior, and Grasha's theory on teaching styles (Leary, 1958; Grasha, 1994; Wubbels et al., 2012). It describes how (teaching) styles are constructed from dominance, friendliness and competence expressions, and the (learning) activities for which they are appropriate.
- The *Feedback* model is based on motivational interviewing techniques that distinguish four informative feedback styles (corrective, descriptive, evaluative or confirmatory responses) and four motivational feedback styles (encouragement, praise, remark or mood matching) (Schunk and Lilly, 1984; Tudge et al., 1996). It specifies the events and states that trigger the corresponding feedback style, and the speech acts for each style.
- The *Explanation* model describes characteristics of the explanations and the agents involved (Neerincx et al., 2018). It distinguishes Roles (such as student and teacher), Explanation Types (such as contrastive and BDI-based), Interpretation, Explanandum, Explanans, One or more statements provided through some medium (e.g., sentences) that are offered to explain a phenomenon or an argument.
- The *Small Talk* model provides the data structure specifications for all kinds of small talk dialogues. It distinguishes Starters, Prompts, Disclosures (with topic and intimacy level) and Closure parts to conduct such dialogues. Other concepts have been added to enrich the conversation, like parameters concerning Intimacy level, Topic, Valence, and Liking (Burger et al., 2017).

**Figure 6:**
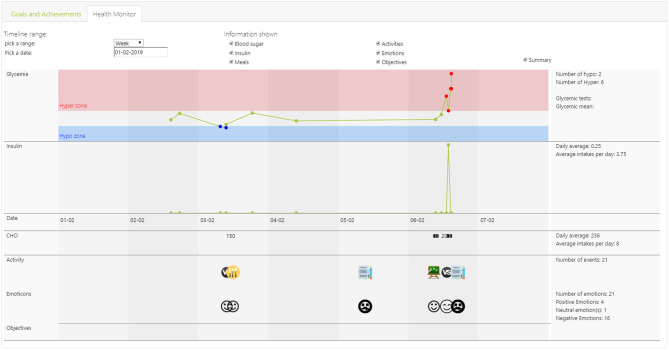
Screenshot of the “Health Monitor” dashboard for Health Care Professionals, showing over a time period: Child's glycemia level (e.g., hypo / hyper), insulin administration, carbohydrates (CHO) in consumed nutrition, activities and emotions.

This ontology represents an important part of the human-robot *collective intelligence*: Knowledge that the robotic system and the humans share and use for their reasoning and conversations (e.g., the feedback and conversations). For example, Figure 6 presents the “Health Monitor” tab of the Health Care Professional's dashboard that is based on the Domain Model, sharing information on child's glucose level (hype / hyper), insulin administration, carbohydrates in consumed nutrition, activities and emotions (The “Goal and Achievements” tab, not shown, contains child's plan and progress in achievements, goals and tasks, based on the PAL Objective Model).

### 4.3. Use Cases, Requirements, and Claims

Following the partnership functions and knowledge-base, we specified more in detail a set of use cases *with* the required PAL functions (i.e., the functional requirements) and expected effects (i.e., the claims; see Figure 1). Each use case refers explicitly to an objective, its pre- and post-conditions and the actors involved. It specifies the sequence of actions and dialogues with an explicit reference to the corresponding requirement and claim. For example, the *use case* “Managing child's objectives” contains an action and dialogue “HCP monitors child's progression at his or her work place,” referring to a *requirement* “PAL shall provide an interactive overview of the realized and active objectives” and a *claim* “HCP identifies progress successes and delays effectively and efficiently.” Use cases have been defined for the hospital and home settings. Requirements have been derived for the overall system, the actor (robot and avatar) behaviors, the timeline, the dashboards, and the tasks. Claims concerned diabetes management behaviors (e.g., the working on the learning goals) and outcomes (e.g., the HbA1c as measure of blood glucose regulation in the last period), well-being indicators (e.g., child vulnerability), PAL system usages (e.g., usage time), and social effects (e.g., responsibility transfer). In total, 19 measuring instruments were selected or constructed to test the claims. Looije et al. (2017) give a detailed description of the method (and tool) to specify and test the use cases, requirements and claims in a coherent way.

## 5. Evaluations of the prototypes

**Figure 7:**
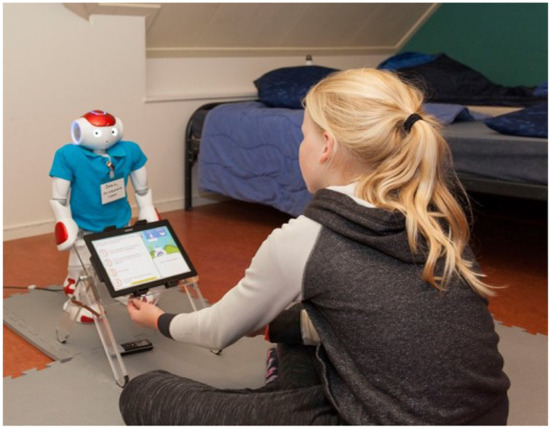
Child-robot interaction during the quiz.

As mentioned before, the 4-year PAL-project entailed three design-test cycles. A summary of the SCE specification per design cycle can be found in Table 1. After the first year, we established the first integrated system that was tested in Italy and the Netherlands. Following the incremental development approach, the system kept on running, being available for all the tests and being updated when appropriate (development was taking place on a test environment, a “copy” of the system in use). This way, the development and evaluation activities could continue in parallel and prototypes could be always assessed at all locations. A diverse set of complementing, formative and summative, evaluations was conducted during each cycle. For the formative studies, we developed the 4C-suite that has been described in section 3.1. Dedicated usability tests were performed, e.g., focusing on (a) the games usage at home, the hospital (e.g., see Figure 7) or diabetes camp, (b) the comprehensibility and use of the objective model, (c) the ease-of-use in general, and (d) the dashboards (e.g., Peters et al., 2019b).

**Table 1:** Explanatory selection of PAL's foundation, core functions, use case (UC) implementations, and claims that were evaluated per design-test cycle.

**Cycle**	**Foundation**	**Core functions**	**UC implementation**	**Claims**
**1**	Self-determination theory, zone of proximal development, gamification, ALIZ-E design rationale. Value stories, journey maps, co-designed scenarios. Cloud computing, hybrid AI and federated ontology.	*R1*: PAL shall provide learn-by-playing activities with personal, reliable, and reinforcing assistance on diabetes management. *R2*: PAL actor shall show empathic partnership. *R3*: PAL shall support joint planning and pursuing personalized objectives.	*Robot interaction*: Acquaintance, quiz. *MyPAL environment*: Avatar, timeline and quiz. *Dashboards*: PAL control and inform.	*C1*: Child has increased knowledge on T1DM. *C2*: Child likes the PAL actor (robot and its avatar). *C3*: Child experiences diabetes-related activities more positively.
**2**	Social penetration theory, motivational interviewing, folk psychology. New co-designed scenarios. System reliability, usability engineering for children.	*R4*: PAL actor shall share experiences via mutual self-disclosure. *R5*: PAL actor shall provide feedback and explanations on behavior. *R6*: PAL actor shall show personalized learning styles	*Robot interaction*: Break and sort game. *MyPAL environment*: Dialogues, reward system (earn coins) and a shop.	*C4*: Child bonds with the PAL actor via the robot *and* its avatar. *C5*: Child is motivated to work on his or her personal objectives with PAL.
**3**	Expert knowledge on child's learning processes for diabetes management with culture- and hospital dependencies. New co-designed scenarios. Game-based learning.	*R1.1*: PAL's support for planning and pursuing objectives shall be personalized and harmonized to child's daily life. *R7*: PAL shall propose and commit on agreements for information sharing.	*PAL actor*: Small talk, dancing designed by child. *MyPAL environment*: Tip of the day, memory games (3), videos, real world tasks, high score board, interactive overview of objectives. *Dashboards*: Making agreements about information sharing.	*C1.1*: Child has increased situated knowledge on T1DM. *C6*: Child is aware of T1DM state and causes and develops self-efficacy *C7*: Child has a higher Quality of Life concerning T1DM *C8*: Children seamlessly follow culture- and hospital-dependent diabetes management processes. *C9*: Child pursues relatively difficult goals.

*The successive rows (cycles) show the increments on the previous row (i.e., the extensions of the foundation, functions, implementations, and claims)*.

In *Cycle 1*, a first version of the human-robot partnership framework was worked out, built and tested. The Self-Determination Theory (SDT) is an important foundation of the objectives that are being served, provided and established by, respectively, the functions, use cases and (expected) effects in the design specification (see Figure 1). For the “gamified” quiz use case with an empathetic robot and avatar (among other things), three claims were specified that fulfill the human basic needs: Increased knowledge for competence (e.g., being able to recognize symptoms of a hypo), liking for relatedness and positive experiences for autonomy. These concepts were applied and tested at diabetes camps and hospitals with children aged 7–10 years. First, during 1-week diabetes camps in Italy and the Netherlands, a user needs assessment was conducted and PAL mock-ups were tested (*N* = 55). Second, an initial version of the PAL system (PAL 1.0) was evaluated in a 1-month test at Italian and Dutch hospitals (*N* = 21). The claims were tested in these different evaluations, for usage periods between 1 and 4 weeks. In general, positive effects were recorded on the SDT-related claims. Children enjoyed to interact with the PAL robot and avatar (which made diabetes-related activities more positive) and showed an increased diabetes knowledge when using the PAL system (i.e., functions *R1 and R2* and claims *C1, C2, and C3* in Table 1). However, for meaningful benefits over a longer period, the PAL system needed substantial improvement. Particularly, shortcomings of the reliability, usability, and goal structure hindered the acceptance and trust of health care professionals. Further, enhanced personalization proved to be needed to establish adherence of the children.

In *Cycle 2*, the SCE-activities continued, building on the results of the first cycle. After establishing the general PAL framework in cycle 1, innovative PAL functions were identified for which, first, the specific module had to be developed and tested, before it would be integrated in the overall system (actually, already in cycle 1 and continuing in cycle 3, this modular approach was taken). For example, Burger et al. (2017) tested the experience sharing function with 11 children over the course of ~ 2 weeks at home (i.e., function *R4* and claim *C4* in Table 1). The number of child disclosures proved to be an indication of their perceived relatedness at the end of the experiment. The higher the relatedness, the better the system usage. Subsequently, this function was implemented in the PAL system. In a similar way, the feedback & explanation functions were tested (i.e., function *R5* and claim *C1* in Table 1). For example, Kaptein et al. (2017) tested robot's self-explanation with 19 children and 19 adults in which the robot performed actions to support type 1 diabetes mellitus management. Adults showed a higher preference for goal-based explanations than children, providing a foundation for personalizing the explanation. The explanations have been integrated in the PAL system. As a third example, Peters et al. (2017b) developed a model of non-verbal warmth and competence robot behaviors, which is expected to improve robot's teaching style (i.e., function *R6* and claim *C1* in Table 1). A perception experiment at primary schools and a diabetes camp showed that even subtle behavior manipulations affect children's warmth-competence perceptions of the robot. This model has been implemented in the PAL system. The last example of a focused experiment concerns the avatar function that was tested at a diabetes camp (Sinoo et al., 2018). The bonding with the physical robot was higher, but this effect reduced when children perceived the physical robot and its avatar more as the same agency. The stronger friendships, the higher the motivation to perform the tasks to do. Therefore, we improved the similarity and consistency between robot and avatar in the next version of PAL. Finally, a study in Italian and Dutch hospitals was conducted with children aged 7–12 years (*N* = 35). The primary aim of the experiment was to refine, further develop and evaluate the second release of the PAL System (2.0), during a longer period of use (i.e., from 3 to 4 months). Main results were that the children bonded with the PAL actor (robot and avatar), and perceived the robot and avatar somewhat as similar. During the experiment, they perceived it increasingly as a buddy who was supporting and making them happy (i.e., functions *R4, R5, and R6* and claim *C4* in Table 1). Notably, the majority of the children stopped using MyPAL App some weeks after the beginning of the study. A large number of children already had participated in cycle 1 and as can be expected, the novelty effect disappeared. They felt there was insufficient new interesting content (i.e., amount and variety of activities) and rather limited child-actor interactions to maintain motivation to use the PAL system for such a long period.

In *Cycle 3*, based on the results of the two first cycles, to work toward ongoing and impacting use of the PAL system by children in regard to T1DM self-management, we introduced new and improved existing functionalities in the PAL system, which were discussed earlier in this paper: (1) General usability of the MyPAL app, goal setting, enriched interaction, additional educational material, gamification, and monitoring for parents (PAL dashboard). This last design-test cycle, contained a randomized controlled trial: A summative evaluation that compared child's self-management with the PAL-system 3.0 vs. “care as usual,” for a period of twice 3 months (with 49 children aged 7–14 years, in the Netherlands and Italy). Phase 1 (the first 3 months) consisted of the effect study, and phase 2 of an implementation study in which both the children who used PAL and the children who got care-as-usual (“waiting-list”) could chose to use a further improved version of PAL (PAL-system 3.5). In total, 14 children interacted with the MyPAL application for 6 months while 26 children participated with the MyPAL application for 3 months (16 were in the waiting-list group and 10 participated in phase 1 only). Each phase started and ended in the hospital. In the intervention condition at the hospital, the child, parent and health care professional set or reflected on the objectives and made agreements about information sharing using the PAL dashboard. Further, during the first visit to the hospital, the interaction with the robot consisted of introduction, acquaintance and play of a game (quiz or break and sort). During the last visit to the hospital, the robot also did a dance choreographed by the child through MyPAL at home (with the avatar). In between the hospital visits, over a 3 month period, the children could play with the avatar via the MyPAL application at home. It is a “real” avatar that continues the activities and interactions of the robot; i.e., the robot and avatar have “only” a different embodiment but act as the same actor (based on the same models and memory). The children were free in deciding how often they wanted to play with the system. Interaction with the avatar at home consisted of saying “hello,” reviewing personal goals, and performing tasks contributing to goals. Human-robot interactions entailed, for example, one of the educative games (quiz, break & sort, memory), watching a video, keeping a diabetes diary, a real life activity, dialogue acts (task suggestions, tips, feedback & explanations) or small talk.

PAL proved to partially support the three human basic needs that affect the development and habituation of human behaviors in a social environment, such as disease self-management (see Self-Determination Theory, section 3.1). Children liked the PAL-robot and were motivated to continue the robot-mediated tasks (*relatedness*). This is consistent with the results of a previous experiment at the diabetes camp, presented in section 5 (Sinoo et al., 2018). The tasks to pursue differed between Italian and Dutch children, reflecting cultural differences on diabetes management (function R1.1 and claim *C8*). In regard to diabetes knowledge, children in the intervention group, using the PAL 3.0, in comparison to the control group, showed a stronger increase after 3 months, than children in the wait-list group [*F*_(30)_ = 4.17, *p* = 0.05]. Moreover, we found a correlation between time playing with the MyPAL app and children's knowledge. Also, children in the intervention groups had a stronger increase in self-care score [*F*_(30)_ = 6.60, *p* = 0.01], as an indication of improved *autonomy*. Furthermore, younger children in the intervention group showed a stronger increase in self-care score, in comparison with their older peers in the intervention group (*p* = 0.03). We did not find an effect of PAL on parental stress and child's glucose regulation (including HbA1c and percentage of measures in healthy range). However, we did find an effect on diabetes related quality of life in children [*F*_(30)_ = 6.14, *p* = 0.02] (i.e., functions *R1 through R7* and claims *C1.1 and C5 through C9* in Table 1). Blanson Henkemans et al. (submitted) provide a detailed description of the randomized controlled trial.

## 6. Conclusions

This paper presented an overview of the SCE-methodology and its application for the PAL research & development activities to develop a robotic partner. As a first contribution to the field of social robotics, it shows how to progressively integrate domain and human factors knowledge into social robots via co-design, modeling and evaluations. The models and design rationale, integrated in the robots, are constructed for re-use and further development. As a second contribution, the paper presents a social robot with dedicated partnership functions and a corresponding knowledge-base that is constructed and shared with the human stakeholders. This robotic system has been evaluated “in the wild,” i.e., at hospitals, diabetes camps and home, in Italy and the Netherlands.

**Figure 8:**
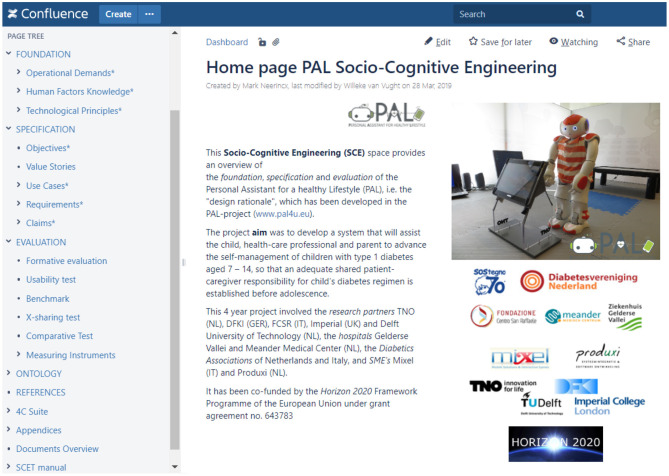
Screenshot of the menu and PAL start page of the Socio-Cognitive Engineering Tool (SCET).

The introduction of this paper distinguished three research questions. Section 2 proposed the Socio-Cognitive Engineering (SCE) methodology as answer to the first question: “How to develop human-agent partnerships for long-term lifestyle support?” We succeeded to integrate into the PAL-system: (a) theories, models and methods from different scientific disciplines, (b) technologies from different fields, (c) diabetes management practices from different nations and hospitals, and (d) last but not least, the diverse individual and context-dependent needs of the children and their caregivers. Our PAL experiences underpin the argumentation for SCE in section 2, but it needs further grounding in usages by others. It should be mentioned that the re-usable PAL design rationale, ontological models and Co-design for Child-Computer Companionship (C4) suite are maintained and accessible in the Socio-Cognitive Engineering Tool (SCET), which is built and maintained in Atlassian Confluence (a wiki content tool for teams to collaborate and share knowledge efficiently; currently within the PAL consortium, but we are exploring ways to share it with other research & development communities). Figure 8 shows a screen shot of the SCET with PAL content (Note that the menu left is consistent with Figure 1).

The second research question was addressed in section 4 and illustrated by Figure 5: “How can a robot partner support the daily diabetes management of children over a longer period?” This section described the 4 partnership functions, the knowledge-base and interaction design of the situated human-robot partnerships for the development of child's disease self-management. In our view, it is one of the first examples of prolonged human-agent/robot teamwork for a healthy lifestyle that has been researched, developed and tested in the field. It represents a new type of evolving human-robot systems with collective intelligence. Both the robot and the human stakeholders acquired more knowledge about child's diabetes management (e.g., recorded in the ontology, like the PAL Objective Model).

The third evaluative question can be answered positively: “Does this partnership improve child's diabetes-control and well-being?” Section 5 provided a brief overview of the evaluation results. PAL proved to support the children on the three basic needs of the Self-Determination Theory: autonomy, competence, and relatedness. To our knowledge, PAL provided the first field study of prolonged “blended” care with a robot for children with a chronic disease, showing positive results in a 3 month evaluation period.

In the next steps of the research and development, we recommend to improve the team aspects concerning responsibility transfer and caregiver involvement. For this, explicit responsibility (transfer) objectives should be included in the PAL Objective Model, and the PAL dashboards should be integrated with the hospital information system (i.e., the work environment of the HCPs). Further, the children would profit substantially from better (technical) integration of their diabetic measurement and administration devices with the PAL system.

Another direction is to apply the models and methods for the management of other diseases of children, such as asthma, and patient or client groups, such as older adults with Type2 Diabetes. Concerning scientific progress, we are researching hybrid AI models that can provide enhanced personalized predictions on patient's health condition (such as hypo or hyper) and can explain these predictions to humans in a way that the human can understand and use (e.g., for the child, the parent, and the HCP).

## Data Availability Statement

The datasets generated for this study will not be made publicly available for the Protection of privacy.

## Ethics Statement

Each study involving human participants has been, from case to case, reviewed and approved by the reference ethical committees of the concerning participating organizations (i.e., the hospitals and research partners in the Netherlands and Italy). Written informed consent to participate in this study was provided by the participants and, when appropriate, participants' legal guardian/next of kin.

## Author Contributions

All authors listed have made a substantial, direct and intellectual contribution to the work, and approved it for publication.

### Conflict of Interest

MN, WV, and OB were employed by the non-profit organization TNO. BK was employed by the non-profit organization DFKI. DF was employed by company Mixel, Italy. BB was employed by company Produxi, The Netherlands. The remaining authors declare that the research was conducted in the absence of any commercial or financial relationships that could be construed as a potential conflict of interest.
